# Efficacy of Three Commercial Disinfectants in Reducing Microbial Surfaces’ Contaminations of Pharmaceuticals Hospital Facilities

**DOI:** 10.3390/ijerph18020779

**Published:** 2021-01-18

**Authors:** Giuseppina Di Martino, Salvatore Pasqua, Bruno Douradinha, Francesco Monaco, Chiara Di Bartolo, Pier Giulio Conaldi, Danilo D’Apolito

**Affiliations:** 1 Unità di Medicina di Laboratorio e Biotecnologie Avanzate, IRCCS-ISMETT (Istituto Mediterraneo per i Trapianti e Terapie ad Alta Specializzazione), Via E. Tricomi 5, 90127 Palermo, Italy; gdimartino@ismett.edu (G.D.M.); bdouradinha@fondazionerimed.com (B.D.); fmonaco@ismett.edu (F.M.); cdibartolo@ismett.edu (C.D.B.); pgconaldi@ismett.edu (P.G.C.); 2 Unità Prodotti Cellulari (GMP), Fondazione Ri.MED c/o IRCCS-ISMETT, Via E. Tricomi 5, 90127 Palermo, Italy; spasqua@fondazionerimed.com; 3 Unità Medicina Rigenerativa ed Immunoterapia, Fondazione Ri.MED c/o IRCCS-ISMETT, Via E. Tricomi 5, 90127 Palermo, Italy

**Keywords:** efficacy, disinfectant, surface contaminations, disinfection procedure, validation

## Abstract

To evaluate and validate the efficacy of disinfectants used in our cleaning procedure, in order to reduce pharmaceutical hospital surfaces’ contaminations, we tested the action of three commercial disinfectants on small representative samples of the surfaces present in our hospital cleanrooms. These samples (or coupons) were contaminated with selected microorganisms for the validation of the disinfectants. The coupons were sampled before and after disinfection and the microbial load was assessed to calculate the Log_10_ reduction index. Subsequently, we developed and validated a disinfection procedure on real surfaces inside the cleanrooms intentionally contaminated with microorganisms, using approximately 10^7^–10^8^ total colony forming units per coupon. Our results showed a bactericidal, fungicidal, and sporicidal efficacy coherent to the acceptance criteria suggested by United States Pharmacopeia 35 <1072>. The correct implementation of our cleaning and disinfection procedure, respecting stipulated concentrations and contact times, led to a reduction of at least 6 Log_10_ for all microorganisms used. The proposed disinfection procedure reduced the pharmaceutical hospital surfaces’ contaminations, limited the propagation of microorganisms in points adjacent to the disinfected area, and ensured high disinfection and safety levels for operators, patients, and treated surfaces.

## 1. Introduction

Microbial contamination of hospital surfaces is a fundamental aspect which requires constant monitoring and control, in order to minimize the transmission of microorganisms from surfaces to patients, to reduce both morbidity and mortality due to nosocomial infections [1,2,3]. 

The emergence of multiple resistant pathogens to several antibiotics has increased the need for effective disinfection. This is especially true in cleanrooms where pharmaceuticals or advanced therapy medicinal products (ATMPs) are prepared. The design, validation and implementation of documented and approved disinfection programs are fundamental for the maintenance of critical areas. The disinfection protocols must be effective and safe for staff, patients, and the surfaces to be cleaned [4]. 

A disinfectant can be defined as a chemical which reduces the number of microorganisms present on a surface. Disinfectants vary in their spectrum of activity, mode of action, and efficacy. The disinfectants currently in use are categorized as non-oxidizing and oxidizing agents. The former includes alcohols, aldehydes, amphoteric compounds, phenolics, and quaternary ammonium compounds, while the latter includes oxygen-releasing compounds such as peracetic acid and hydrogen peroxide [5,6]. 

The first step of an efficient disinfection program is the choice of disinfectants that guarantee bactericidal, fungicidal, and sporicidal actions, as previously demonstrated by the manufacturer in compliance with United States Pharmacopoeia (USP) 35 Chapter <1072> [7]. For our disinfection procedures, we chose 6% hydrogen peroxide, quaternary ammonium, and 70% isopropanol based commercial disinfectants. We developed an in-house quantitative analytical disinfection method to test their efficacy to eliminate different microorganisms spread over multiple types of surfaces in cleanrooms. We also considered the feasibility and the costs associated with sampling. Over the last decades, protocols for the recovery of microorganisms from surfaces often led to variable results due to differences between sampling tools, individual operators, low microbial recovery rates, and low levels of reproducibility [8]. To minimize the experimental variability between the disinfectant efficacy test done in the microbiological laboratory and the one performed for validation of the disinfection procedure in the controlled areas, we performed the recovery of microorganisms as recommended for environmental sampling of flat surfaces. Therefore, we chose to do the surface coupon testing with minor modifications, i.e., avoiding the use of swabs and creating a support for the coupons. This allows the recovery of the microorganisms and the replication of potential contaminations present in the coupons directly into the contact plates containing the disinfectants’ inactivating agents.

Using our method, we were able to detect all tested suspensions of microorganisms present on the coupons’ surfaces and to evaluate the activity of the chosen disinfectants, demonstrating the efficacy of the latter to efficiently eliminate the microorganisms, as required by USP 35 <1072> [7], which specify that disinfectants must have a bactericidal, fungicidal, and sporicidal activity, showing a Log_10_ reduction of three for bacteria and fungi and of two for spores. To demonstrate the effects induced by several environmental factors, like temperature, pH, detergent residues, mechanical stress, and surface attachment in the facility, we validated a disinfection procedure in situ, using the microbial species required by USP 35 <1072> [7] and three additional potential contaminants of our Good Manufacturing Practices (GMP) facility [9].

Our results showed that the proposed in-house analytical method was efficient for confirming the tested disinfectants’ efficacy. Moreover, we observed that all chosen disinfectants used for the validation of our cleaning procedure were suitable for surface disinfection, since they reduced and controlled the spread of contamination in surfaces representative of hospital sensitive areas.

## 2. Materials and Methods

### 2.1. Microbial Species Required by USP 35 <1072> and In-House GMP Facility Strains

American Type Culture Collection strains (ATCC; Table 1) were purchased from Microbiologics (St. Cloud, MN, USA) and supplied as quantitative microorganism lyophilized pellets. For each strain, a microbial suspension was prepared according to the manufacturer’s instructions to obtain a mother suspension with a concentration of 10^7^ ≤ colony forming units (CFU)/mL < 10^8^.

In-house GMP Facility strains (Table 1), isolated from previous environmental monitoring in our facility, were utilized with not more than five passages from the original culture [9] and grown in tryptic soy broth (TSB; purchased from BD, Franklin Lakes, NJ, USA) until they reached the early exponential phase. The culture concentration was adjusted to 0.5 based on the McFarland scale (around 10^8^ CFU/mL).

### 2.2. Microorganism Suspension Preparation and Titer Determination

Seven serial decimal dilutions from the mother dilution were prepared in peptone water (BD). To assess viability, purity, and titer determination, 100 μL of each dilution were plated on tryptic soy agar (TSA, for bacterial suspensions) and Sabouraud dextrose agar (SDA, for fungal suspensions) plates containing lecithin and polysorbate 80 (both from BD) and incubated at 37 °C. The CFUs were counted after 2–3 days (bacteria) and 4–5 days (yeast and mold). The titer was determined by calculating the weighted arithmetic average of the CFU number for each dilution. 

### 2.3. Disinfectants

The disinfectants used in this study were all ready-to-use Steri-perox 6% (6% hydrogen peroxide formulated with 94.0% USP water for injection, purchased from Veltek Associates Inc., Malvern, PA, USA), Dec-quat 100 (based in quaternary ammoniums alkyl dimethyl benzyl ammonium chloride and alkyl dimethyl ethyl benzyl ammonium chloride; Veltek Associates Inc.), and Dec-ahol (based in 70% isopropyl alcohol formulated with 30% USP water for injection; Veltek Associates Inc.). All 3 products were tested for bactericidal and fungicidal activity. Steri-perox 6% was also tested for sporicidal activity.

### 2.4. Preparation of Coupons

The surfaces present in the pharmaceutical cleanrooms were the following: glass, AISI 304 steel, PVC, melamine, and compact polycarbonate. For each material type, coupons were prepared with dimensions of 6 × 6 cm. Each square was glued using a 3M adhesive over the lid of a 50 mL tube. The coupons and tubes were sterilized in a tyvec bag (Johnson & Johnson, New Brunswick, NJ, USA) in a STERRAD sterilizer, using hydrogen peroxide gas plasma for 54 min at 45 °C. The sterilization efficiency was evaluated by the color change of the indicator strips present in the tyvec bags and the biological indicators “Sterrad Cyclesure” (Johnson & Johnson), and incubated for 24 h at 37 ± 2 °C.

### 2.5. Analytical Method and Experimental Protocol to Evaluate Disinfectants’ Efficacy

**Figure 1:**
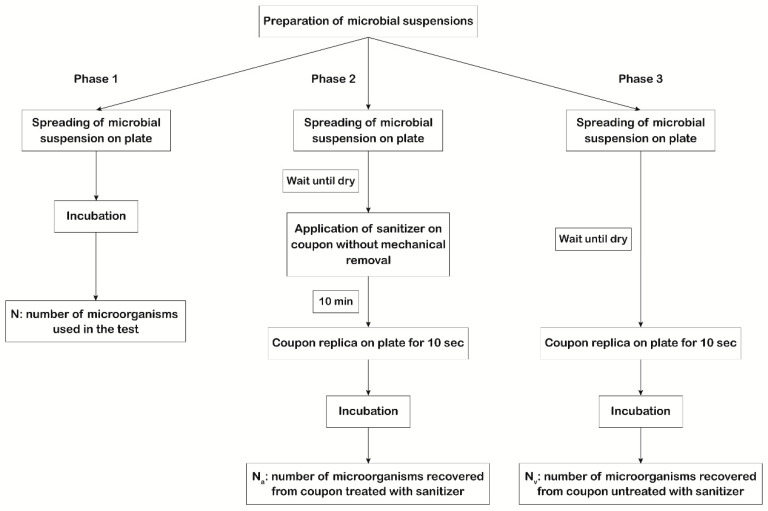
Scheme of the experimental plan used to evaluate disinfectants’ efficacy. In phase 1, 100 μL of each microbial suspension was plated in triplicate, and for each microorganism, the titer of the mother suspension was determined (N). In phase 2, 100 μL of each microbial suspension was applied, in triplicate, on the coupon surface with a spreader. After drying, the disinfectant was applied without mechanical removal (worst case scenario). After 10 min of action, sampling of the coupon surface was performed by direct replication on plates to determine the number of vital microorganisms that survived the action of the disinfectant (N_a_). In phase 3, 100 μL of each microbial suspension was applied, in triplicate, on the coupon surface with a spreader. After drying, sampling of the coupon surface was performed by direct replication on plates to determine the number of vital microorganisms dispensed on the coupon surface (N_v_).

The first scope of our study was to evaluate the efficiency of the 3 types of disinfectants mentioned above by measuring their action against the microorganisms suggested by USP 35 <1072> [7] spread on the coupons. The experimental protocol involved testing the action of each disinfectant on 6 × 6 cm coupons contaminated with titrated microbial suspensions. The experimental plan (Figure 1) involved three phases to be performed in parallel using the same microbial suspensions.

The recovery of microorganisms was performed using TSA or SDA plates containing lecithin and polysorbate 80 (BD) for bacteria/spores and fungi, respectively. The use of lecithin and polysorbate 80 prevented the sanitizer’s carryover inhibition [10]. The first phase regards the evaluation of the viability, purity, and titer of microbial suspensions. One hundred microliters of each microbial suspension were plated in triplicate, and for each microorganism, the titer of the mother suspension was determined (N). In the second phase, the number of microorganisms that survived the action of the disinfectant on the coupon surface (N_a_) was determined by applying, in triplicate, one hundred microliters of each microbial suspension on the coupons with a spreader. After drying, the disinfectant was applied without mechanical removal (considered the worst case scenario) and allowed to act for 10 min, as suggested by the manufacturer. After drying, sampling of the coupon surface was done by direct replication on plates, to asses N_a_. In the third phase, the number of vital microorganisms dispensed on the coupon surface (N_v_) was quantified by spreading, in triplicate, one hundred microliters of each microbial suspension on the coupons. After drying, sampling of the coupon surface was performed by direct replication on plates.

### 2.6. Log_10_ Reduction Index and Acceptance Criteria

The efficacy of the disinfection action was determined by calculating the Log_10_ reduction index. Log_10_ reduction = Log_10_ (N_v(Msusp)_/N_a(Msusp)_).

The formulas applied are the following:

- N_a(Msusp)_ = N_a_ × DF × V
- N_v(Msusp)_ = N_v_ × DF × V

in which DF is the dilution factor of the first dilution, taken into consideration for the calculation of the weighted average; and V is the suspension volume inoculated on a plate (100 µL). Subsequently the Log_10_ reduction index was calculated as stated above. The acceptance criteria, in accordance with USP 35 <1072> [7] and also declared by the disinfectants’ manufacturer, were a Log_10_ reduction ≥3, in the case of bactericidal/fungicidal activity; and a Log_10_ reduction ≥ 2, in the case of sporicidal activity.

### 2.7. Microorganisms Identification

For each step of the experiments, the purity of each microorganism was determined by microscope observation and gram staining, and species identification was performed using Vitek MS (Biomerieux, Marcy-l’Étoile, France), according to manufacturer’s instructions.

### 2.8. Robustness

An analytic method is considered robust when it is not influenced by small but intentional variations in method parameters or usual conditions, e.g., different days, materials, or users, and gives an indication of its reliability during normal use. To evaluate the robustness of our disinfection procedure, the experiments described below were performed 3 times in 3 different days by 3 different operators.

### 2.9. Cleaning and Disinfection Standard Operating Procedure

Subsequently, the study included the design of the standard operating procedure (SOP), the theoretical and practical training of the personnel, and the final validation of the SOP. The aspects taken into consideration to write the SOP were the materials to be used, the method of application of disinfectants, and their rotation, to avoid the onset of potential resistance. The experimental protocol includes intentional contamination of the surfaces present in controlled areas, with approximately 10^7^–10^8^ CFU (worst case scenario: heavy contamination of surfaces) of reference microorganisms and in-house GMP facility strains (Table 1). For our study, we also used *Kocuria rosea*, *Staphylococcus epidermidis,* and *Micrococcus luteus*, which are typical of cleanrooms’ microbiota [11]. The surfaces were subjected to sampling to evaluate the efficacy, reproducibility, and robustness of our cleaning and disinfection procedure. Accordingly, we decided to perform a weekly rotation program of Steri-perox 6% and Dec-quat 100. Furthermore, for all steel or glass surfaces and for all those on which the pharmaceutical product is prepared, an extra final step of disinfection with Dec-ahol was done, in order to remove any traces of the first disinfectant, thus ensuring a higher level of disinfection and greater safety for patients and surfaces. The disinfectants were applied on the surface using polyester sterile wipes. The steps to follow were:

(1) Saturation of the wipes by immersion in the first disinfectant solution
(2) Squeezing the wipes to remove excess liquid;
(3) Passing the wipe over the surface, proceeding from top to bottom and from left to right, overlapping the passes and making sure that there were no areas where the wipe has not been passed;
(4) Contact time of action of disinfectant was 10 min;
(5) For the surfaces described above, repetition of the first 4 points with the Dec-ahol.

### 2.10. Validation of Cleaning and Disinfection Procedure

At the beginning of validation procedure, the rooms were initially cleaned and subjected to sterilization using vapor phase hydrogen peroxide (VPHP) by a specialized company (Belsar Srl, Tradate, Italy) thoroughly, to eliminate any potential contaminations. The coupons that were contaminated were representative of the surfaces. For each type of material, we selected 3 areas of 4 × 4 cm. These dimensions allowed us to directly sample the entire surface with the Replicate Organism Detection and Counting (RODAC) contact plates (BD), specifically for environmental monitoring. Each of these areas were contaminated with approximately 10^7^–10^8^ CFU of the microorganisms detailed in Table 1. The procedure was performed blindly to all surfaces, e.g., one operator contaminated specific surface points, while another would clean the surfaces homogeneously without knowing which areas had been contaminated. Subsequently, the trained personnel did the environmental sampling using TSA or SDA RODAC contact plates, containing lecithin and polysorbate 80. In addition, to confirm that spreading of the microorganisms to the areas adjacent to those contaminated did not occur, we also sampled the surrounding area [12]. The number of total CFU that survived the cleaning procedure was determined by calculating the arithmetic average of the values obtained in the 3 plates in the same experiment. As before, the validation procedure was repeated 3 times by 3 different operators on three different days to confirm repeatability and robustness of the assay [13]. The results obtained for the 3 experiments are expressed as the Log_10_ reduction of the average value of the 9 experiments performed, for each microorganism. After validation, the rooms were subjected to sterilization using VPHP. 

## 3. Results

### 3.1. Microorganisms Identification and Purity

For each step of the experiments, a representative colony of the microorganisms recovered from the sampled surfaces was subjected to identification by Vitek MS to confirm its identity, purity, and correspondence to the original suspension inoculated on the coupons. All samples were confirmed as the ones applied to the surface, and no cross-contaminations were found (data not shown).

### 3.2. Disinfectants’ Efficacy and Log_10_ Reduction Index

Log_10_ reduction values were obtained by evaluating the disinfectants efficiency over the reduction of microorganisms’ viability on each tested surface (Table 2). *Aspergillus brasiliensis* was consistently the most resistant to both quaternary ammonium compound and isopropyl alcohol. Our results were consistent with the acceptance criteria required by USP 35 <1072> [7].

### 3.3. Validation of Cleaning and Disinfection Procedure

Our cleaning and disinfection procedure was performed as described above. Environmental sampling results, expressed as Log_10_ reduction indices, are summarized in Table 3.

These results showed that our disinfectant application procedure is able to routinely eliminate the tested microorganisms on the materials present in the production area. Furthermore, the use of Steri-perox 6% and Dec-ahol, or Dec-quat 100 and Dec-ahol, on the critical surfaces where the pharmaceutical drugs are produced almost completely removed the microorganisms present on the surfaces, including the three in-house GMP facility strains, thus demonstrating the efficiency of our disinfection program. Additionally, the sampling of points near to those of the validation showed the complete absence of microorganisms, confirming that the disinfectant procedure does not lead to microorganisms’ diffusion to the adjacent areas. 

### 3.4. Robustness

During the validation period, the rooms were cleaned once daily. We obtained similar and robust results for all tested conditions (Table 4), thus demonstrating that the proposed cleaning procedure eliminates almost all surface contaminations without showing differences that might have been induced by either different operators or different execution days.

The robustness was also confirmed by the trend analysis of environmental monitoring data obtained over three years, which showed that our procedure mantains environmental contamination below the limits set by GMP guidelines, even in extreme situations such as extraordinary maintenance or restructuring, with no signs of corrosion on the treated surfaces.

## 4. Discussion

Nowadays, effective cleaning and disinfection procedures are required due to an increase in the number of microorganisms’ strains which display resistance to multiple antibiotics. Such procedures are important for cleanrooms designed to handle products which will be in contact with patients, especially those under an immunosuppression regimen. Contamination of medical environments with a range of pathogens may lead to undesirable healthcare-associated infections. Several pathogens can survive on surfaces for extended periods and could be disseminated in the hospital via healthcare workers who have direct contact with infected patients and contaminated environmental surfaces [14]. The choice of sanitizing agents is fundamental and must take into consideration their ability to destroy microorganisms, user safety, application method, instrument compatibility, minimization of the induction of bacterial resistance, and their ability to avoid corrosion of materials subjected to disinfection and reduce associated costs [1,6,9,14]. The disinfectants on the market are generally alcohols, chlorine, aldehydes, peroxygenes, and quaternary ammonium compounds [6], and each of them have advantages and disadvantages. Alcohol is cheap and highly inflammable, with a rapid bactericidal effect without bacteriostatic action and relevant toxicity issues, but it is not sporicidal and has a poor efficacy to inactivate some viruses. Chlorine-based disinfectants are widely used in hospital disinfection procedures and show a vast bactericidal spectrum. However, they may cause corrosion of hospital equipment and of metal materials, leading to additional costs for their maintenance and eventual replacement. Moreover, such disinfectants can be easily inactivated by organic matter, cause irritation and burning in several organs, such as skin, eyes, and mucous membranes [6,15]. Hydrogen peroxide is a disinfectant with a good sporicidal action, and due to its rapid action, it is desirable from an environmental point of view. In recent years, the development of accelerated hydrogen peroxide (AHP) has made it possible to achieve higher compatibility with materials, increasing its use for disinfection procedures. An excellent compound with fast action at low concentrations towards all microorganisms and spores is peracetic acid. It is not affected by the presence of organic substances and does not leave residual matter, but it is unstable and has a corrosive effect on metals [16,17]. Together with chlorine-based compounds, quaternary ammonium-based disinfectants are the most commonly used in hospital disinfection procedures. They have a broad spectrum of action towards many microorganisms and sporostatic activity, but exhibit low efficacy against gram-negative bacteria and non-enveloped viruses [15].

Disinfectants can be applied with or without mechanical action. The latter, for example, spray disinfection, is an easy method but causes relatively high atmospheric concentrations of some of the disinfectant components. The former option ensures removal of the vast majority of organic debris or dirt present that could hinder the disinfecting action. The following methods are based in mechanical application: “Spray and Wipe”, “Dip and Wipe”, and “Soak and Wipe”. The “Spray and Wipe” method requires the disinfectant to be sprayed directly onto the surface. However, there are several drawbacks, such as possible over-spraying and the possibility of the disinfectant being inhaled by workers and patients [18]. The “Dip and Wipe” method requires a dry wipe to be immersed in a disinfectant solution for 5–10 s, squeezed to remove excess disinfectant, and used to evenly distribute the liquid over the surface. It is important that the wipe is soaked in an adequate manner to prevent it from losing its antimicrobial activity and becoming a potential vehicle for the transmission of pathogens itself [19]. The “Soak and Wipe” method is similar to “Dip and Wipe” and requires that the wipe remain immersed in a disinfectant solution from 10 min to 8 h. The wipe is then used as described above. This method allows a contact time of the wipe with the disinfectant to ensure sufficient wetting of the fabric before use. However, it was reported that longer soaking led to a decrease in the antimicrobial activity of the disinfectant [20]. A particular “Soak and Wipe” method uses ready-to-use disinfecting wipes distributed by the manufacturer in sealed packs. They are timesaving and ensure convenient implementation and practical and reliable performance. However, their long shelf-life could increase the probability of loss of antimicrobial activity due to the degradation of the disinfectant or interactions between the wipe material and active ingredients, with a risk of increasing healthcare-associated infections [21].

In order to define a cleaning and disinfection procedure capable of keeping the microbial surface contaminations under the limits set by the respective guidelines that was safe for operators, patients, and equipment, and was also economically sustainable, we chose to use three ready-to-use commercial disinfectants to be applied with the “Soak and Wipe” method to limit potential harmful aerosols, reduction of preparation time, and distribute the disinfectants evenly on all surfaces.

The efficacy of our method showed that for all three selected disinfectants, when applied for 10 min on all tested surfaces, a Log_10_ reduction index was obtained that was consistent with the acceptance criteria expressed in USP 35 <1072> and in agreement with the technical specifications from the manufacturer. These results demonstrated the bactericidal and fungicidal efficacy of the three commercial disinfectants chosen for the disinfection of clean rooms. Moreover, Steri-perox 6% also showed sporicidal activity. Furthermore, our protocol based on direct sampling, i.e., without the help of swabs, allowed robust and reproducible results and was more cost-effective in respect to the procedures that used alternative recovery methods [1,9]. These results were useful for estimating the validity of the procedural disinfection parameters and for developing the SOP for the disinfection of pharmaceutical surfaces.

The next aim of the work was to validate the disinfection procedure when applied under practical conditions. Accordingly, we initially wrote a disinfection procedure with the goal of maximizing disinfection performance and minimizing the presence or spread of both microbial contaminations and resistance to the disinfectants. We have chosen to rotate the disinfectants weekly and to apply them by the “Soak and dry” method. Our disinfection protocol involved the sequential use of two different disinfectants, in particular, Steri-perox 6% or Dec-quat 100, followed by Dec-ahol. The validation of the procedure, through in situ studies done on surfaces contaminated with 10^7^–10^8^ CFU (worst case scenario) of the 10 microorganisms detailed in Table 1, have demonstrated strong reduction in the surfaces’ contamination, with at least 6 Log_10_ reduction for all bacteria, fungi, and spores used (Table 3) and no propagation of microorganisms to the points adjacent to the disinfected area.

The results derived from our in situ study validated our disinfection procedure, confirming that this practical approach is able to reduce the microbial surface contaminations. Furthermore, the continuous environmental monitoring of the surfaces over a period of three years has shown that our procedure is safe for the operators who perform the cleaning and keeps the environmental contamination below the stipulated limits. Moreover, the routine inspection of the surfaces showed no signs of corrosion, confirming that the three commercial disinfectants used in our cleaning procedures are safer than other disinfectants, i.e., chlorine based solutions [22]. In addition, these disinfectants are not based on chlorine or phenol, making them less dangerous to personnel, surfaces, and to the environment [18].

## 5. Conclusions

In conclusion, our validation protocol is an example of an efficient and reliable method which evaluates the efficacy and safety of disinfectants in the materials commonly present in cleanrooms. Together with our disinfection procedure, it can be easily applied in different hospital settings, limiting the propagation of microorganisms and maintaining high levels of safety for operators, patients, and integrity of surfaces.

## Figures and Tables

**Table 1:** Microbial strains.

Reference Strains	In-House GMP Facility Strains
*Candida albicans* ATCC 10231	*Kocuria rosea*
*Aspergillus brasiliensis* ATCC 16404	*Staphyloccus epidermidis*
*Staphylococcus aureus* ATCC 6538	*Micrococcus luteus*
*Pseudomonas aeruginosa* ATCC 15442	
*Escherichia coli* ATCC 8739	
*Bacillus subtilis* ATCC 6633	
*Bacillus subtilis spore* ATCC 6633	

**Table 2:** Log_10_ reduction index measured by disinfectants’ efficacy test method. The tests were performed using a contact time of 10 min. Each test was done in triplicate on three different days. The Log_10_ reduction index was calculated. The acceptance criteria, in accordance with USP 35 <1072>, were a Log_10_ reduction ≥ 3, in the case of bactericidal/fungicidal activity; and a Log_10_ reduction ≥ 2, in the case of sporicidal activity.

6 Hydrogen Peroxide	Microorganisms						
**Surface**	**Log_10_ ± St. Dev.**						
	*B. subtilis*	*S. aureus*	*Spores B. subtilis*	*P. aeruginosa*	*E. coli*	*C. albicans*	*A. brasiliensis*
Glass	4.46 ± 0.01	4.52 ± 0.01	2.64 ± 0.08	4.59 ± 0.01	4.64 ± 0.01	4.47 ± 0.01	4.11 ± 0.01
AISI 307 Steel	4.49 ± 0.01	4.41 ± 0.01	2.66 ± 0.06	4.51 ± 0.01	4.58 ± 0.01	4.50 ± 0.01	4.09 ± 0.03
PVC	4.50 ± 0.01	4.44 ± 0.01	2.62 ± 0.07	4.66 ± 0.01	4.58 ± 0.01	4.50 ± 0.01	4.07 ± 0.03
Melamine	4.49 ± 0.01	4.44 ± 0.01	2.68 ± 0.05	4.58 ± 0.01	4.63 ± 0.01	4.49 ± 0.01	4.20 ± 0.02
Compact Policarbonate	4.47 ± 0.01	4.44 ± 0.01	2.64 ± 0.04	4.63 ± 0.01	4.59 ± 0.01	4.45 ± 0.01	4.18 ± 0.02
**Quaternary ammonium**	**Microorganisms**						
**Surface**	**Log_10_ ± St. Dev.**						
	*B. subtilis*	*S. aureus*	*Spores B. subtilis*	*P. aeruginosa*	*E. coli*	*C. albicans*	*A. brasiliensis*
Glass	4.24 ± 0.02	4.38 ± 0.02	-	4.50 ± 0.01	4.50 ± 0.01	4.24 ± 0.02	3.87 ± 0.03
AISI 307 Steel	4.28 ± 0.02	4.36 ± 0.02	-	4.49 ± 0.00	4.48 ± 0.01	4.36 ± 0.02	3.90 ± 0.01
PVC	4.30 ± 0.02	4.41 ± 0.01	-	4.58 ± 0.01	4.49 ± 0.01	4.28 ± 0.02	3.97 ± 0.02
Melamine	4.38 ± 0.02	4.40 ± 0.02	-	4.46 ± 0.01	4.49 ± 0.01	4.34 ± 0.02	3.91 ± 0.04
Compact Policarbonate	4.23 ± 0.02	4.42 ± 0.00	-	4.60 ± 0.01	4.47 ± 0.01	4.36 ± 0.02	3.99 ± 0.04
**70 Isopropanol**	**Microorganisms**						
**Surface**	**Log_10_ ± St. Dev.**						
	*B. subtilis*	*S. aureus*	*Spores B. subtilis*	*P. aeruginosa*	*E. coli*	*C. albicans*	*A. brasiliensis*
Glass	4.18 ± 0.02	4.26 ± 0.01	-	4.35 ± 0.02	4.31 ± 0.02	4.20 ± 0.02	3.84 ± 0.05
AISI 307 Steel	4.24 ± 0.02	4.33 ± 0.02	-	4.37 ± 0.02	4.36 ± 0.02	4.19 ± 0.02	3.82 ± 0.03
PVC	4.25 ± 0.01	4.30 ± 0.02	-	4.52 ± 0.01	4.35 ± 0.02	4.20 ± 0.01	3.92 ± 0.04
Melamine	4.36 ± 0.02	4.25 ± 0.02	-	4.40 ± 0.00	4.38 ± 0.02	4.25 ± 0.01	3.84 ± 0.05
Compact Policarbonate	4.19 ± 0.03	4.39 ± 0.01	-	4.49 ± 0.01	4.45 ± 0.01	4.21 ± 0.02	3.83 ± 0.05

Sporicidal activity was determined only for 6% Hydrogen Peroxide.

**Table 3:** Log_10_ reduction index of surface contaminations determined after the disinfection procedure. Prior to the disinfection procedure, surfaces were contaminated with 10^7^–10^8^ colony forming units (CFU)/mL. For each disinfectant, the contact time was 10 min.

6% Hydrogen peroxide IPA 70%	Microorganisms									
**Surface**	**Log_10_ ± St. Dev.**									
	*B. subtilis*	*S. aureus*	*Spores B. subtilis*	*P. aeruginosa*	*E. coli*	*C. albicans*	*A. brasiliensis*	*K. rosea*	*S. epidermidis*	*M. luteus*
Glass	7.40 ± 0.16	7.40 ± 0.22	6.74 ± 0.09	7.40 ± 0.13	7.40 ± 0.16	7.40 ± 0.16	7.70 ± 0.26	7.22 ± 0.11	7.22 ± 0.12	7.40 ± 0.15
AISI 307 Steel	7.22 ± 0.19	7.70 ± 0.30	6.66 ± 0.08	7.40 ± 0.20	7.70 ± 0.32	7.70 ± 0.34	7.40 ± 0.13	7.40 ± 0.10	7.40 ± 0.15	7.22 ± 0.16
PVC	7.40 ± 0.27	7.40 ± 0.23	6.70 ± 0.08	7.70 ± 0.30	7.70 ± 0.23	7.70 ± 0.30	7.70 ± 0.30	7.22 ± 0.09	7.40 ± 0.18	7.40 ± 0.20
Melamine	7.22 ± 0.21	7.40 ± 0.20	6.66 ± 0.07	7.40 ± 0.26	7.70 ± 0.28	7.70 ± 0.20	7.70 ± 0.26	7.40 ± 0.10	7.22 ± 0.12	7.22 ± 0.16
Compact Policarbonate	7.70 ± 0.30	7.22 ± 0.21	6.66 ± 0.07	7.70 ± 0.23	7.70 ± 0.28	7.70 ± 0.30	7.40 ± 0.15	7.22 ± 0.12	7.10 ± 0.11	7.40 ± 0.15
**Quaternary ammonium IPA 70%**	**Microorganisms**									
**Surface**	**Log_10_ ± St. Dev.**									
	*B. subtilis*	*S. aureus*	*Spores B. subtilis*	*P. aeruginosa*	*E. coli*	*C. albicans*	*A. brasiliensis*	*K. rosea*	*S. epidermidis*	*M. luteus*
Glass	6.80 ± 0.13	6.74 ± 0.14	6.52 ± 0.10	6.70 ± 0.16	6.70 ± 0.09	6.74 ± 0.11	6.74 ± 0.11	6.70 ± 0.10	6.70 ± 0.11	6.66 ± 0.08
AISI 307 Steel	6.66 ± 0.09	6.74 ± 0.12	6.66 ± 0.09	7.22 ± 0.16	6.74 ± 0.10	6.80 ± 0.16	6.74 ± 0.11	6.70 ± 0.09	6.66 ± 0.07	6.70 ± 0.09
PVC	6.80 ± 0.14	6.80 ± 0.16	6.49 ± 0.07	6.74 ± 0.07	6.80 ± 0.12	6.92 ± 0.17	6.80 ± 0.12	6.74 ± 0.12	6.74 ± 0.09	6.70 ± 0.08
Melamine	6.74 ± 0.12	6.80 ± 0.14	6.62 ± 0.11	6.74 ± 0.09	6.74 ± 0.11	6.74 ± 0.14	6.74 ± 0.09	6.74 ± 0.10	6.74 ± 0.08	6.70 ± 0.07
Compact Policarbonate	6.74 ± 0.09	6.74 ± 0.15	6.49 ± 0.11	6.74 ± 0.10	6.74 ± 0.11	6.80 ± 0.13	6.80 ± 0.09	6.70 ± 0.13	6.66 ± 0.07	6.70 ± 0.10

**Table 4:** Surface contamination determined after disinfection procedure. The tests were performed under practical conditions. For each disinfectant, we used a contact time of 10 min. Each test was done by three operators in triplicate on three surfaces. Each operator worked on different days, for a total of nine replicates. Results are presented as the arithmetic mean of the CFU obtained in the nine samplings ± standard deviation.

6% Hydrogen peroxide + IPA 70%	Microorganisms ^a^									
**Surface**	*B. subtilis*	*S. aureus*	*Spores B. subtilis*	*P. aeruginosa*	*E. coli*	*C. albicans*	*A. brasiliensis*	*K. rosea*	*S. epidermidis*	*M. luteus*
Glass	2 ± 0.9	2 ± 1.3	9 ± 2.2	2 ± 0.7	2 ± 0.9	2 ± 0.9	1 ± 0.8	3 ± 0.9	3 ± 1	2 ± 0.8
AISI 307 Steel	3 ± 1.6	1 ± 1	11 ± 2.1	2 ± 1.2	1 ± 1.1	1 ± 1.2	2 ± 0.7	2 ± 0.5	2 ± 0.8	3 ± 1.3
PVC	2 ± 1.7	2 ± 1.4	10 ± 1.9	1 ± 1	1 ± 0.7	1 ± 1	1 ± 1	3 ± 0.7	2.4 ± 1	2 ± 1.2
Melamine	3 ± 1.9	2 ± 1.2	11 ± 1.9	2 ± 1.6	1 ± 0.9	1 ± 0.6	1 ± 0.8	2 ± 0.5	2.7 ± 1	3 ± 1.3
Compact Policarbonate	1 ± 1	3 ± 1.9	11 ± 2	1 ± 0.7	1 ± 0.9	1 ± 1	2 ± 0.8	3 ± 1	4 ± 1.2	2 ± 0.8
**Quaternary ammonium + IPA 70%**	**Microorganisms ^a^**									
**Surface**	*B. subtilis*	*S. aureus*	*Spores B. subtilis*	*P. aeruginosa*	*E. coli*	*C. albicans*	*A. brasiliensis*	*K. rosea*	*S. epidermidis*	*M. luteus*
Glass	8 ± 2.7	9 ± 3.4	15 ± 4	10 ± 4.5	10 ± 2.2	9 ± 2.5	9 ± 2.6	10 ± 2.6	10 ± 2.8	11 ± 2.2
AISI 307 Steel	11 ± 2.5	9 ± 2.9	11 ± 2.5	3 ± 1.3	9 ± 2.3	8 ± 3.5	9 ± 2.6	10 ± 2.3	11 ± 2	10 ± 2.3
PVC	8 ± 3.1	8 ± 3.6	16 ± 3	9 ± 1.6	8 ± 2.6	6 ± 2.8	8 ± 2.5	9 ± 3	9 ± 2.2	10 ± 2
Melamine	9 ± 3	8 ± 3	12 ± 3.5	9 ± 2.1	9 ± 2.6	9 ± 3.4	9 ± 2.2	9 ± 2.3	9 ± 1.8	10 ± 1.7
Compact Policarbonate	9 ± 2.1	9 ± 3.7	16 ± 4.5	9 ± 2.4	8 ± 2.5	8 ± 2.9	8 ± 1.8	10 ± 3.5	11 ± 2	10 ± 2.7

^a^ CFU mean ± standard deviation.

## Data Availability

Data are available on request to corresponding author.
